# Niche Partitioning in Sympatric *Gorilla* and *Pan* from Cameroon: Implications for Life History Strategies and for Reconstructing the Evolution of Hominin Life History

**DOI:** 10.1371/journal.pone.0102794

**Published:** 2014-07-23

**Authors:** Gabriele A. Macho, Julia A. Lee-Thorp

**Affiliations:** Research Laboratory for Archaeology, Oxford, United Kingdom; Max Planck Institute for Evolutionary Anthropology, Germany

## Abstract

Factors influencing the hominoid life histories are poorly understood, and little is known about how ecological conditions modulate the pace of their development. Yet our limited understanding of these interactions underpins life history interpretations in extinct hominins. Here we determined the synchronisation of dental mineralization/eruption with brain size in a 20^th^ century museum collection of sympatric *Gorilla gorilla* and *Pan troglodytes* from Central Cameroon. Using δ^13^C and δ^15^N of individuals’ hair, we assessed whether and how differences in diet and habitat use may have impacted on ape development. The results show that, overall, gorilla hair δ^13^C and δ^15^N values are more variable than those of chimpanzees, and that gorillas are consistently lower in δ^13^C and δ^15^N compared to chimpanzees. Within a restricted, isotopically-constrained area, gorilla brain development appears delayed relative to dental mineralization/eruption [or dental development is accelerated relative to brains]: only about 87.8% of adult brain size is attained by the time first permanent molars come into occlusion, whereas it is 92.3% in chimpanzees. Even when M1s are already in full functional occlusion, gorilla brains lag behind those of chimpanzee (91% versus 96.4%), relative to tooth development. Both bootstrap analyses and stable isotope results confirm that these results are unlikely due to sampling error. Rather, δ^15^N values imply that gorillas are not fully weaned (physiologically mature) until well after M1 are in full functional occlusion. In chimpanzees the transition from infant to adult feeding appears (a) more gradual and (b) earlier relative to somatic development. Taken together, the findings are consistent with life history theory that predicts delayed development when non-density dependent mortality is low, i.e. in closed habitats, and with the “risk aversion” hypothesis for frugivorous species as a means to avert starvation. Furthermore, the results highlight the complexity and plasticity of hominoid/hominin development.

## Introduction

Resource limitation and mortality are key factors that determine life history strategies in mammals [1], including hominoids [2]. Assessing the relative influence of each of these factors has however been found difficult. In (palaeo)anthropology, this problem is further compounded as life history reconstruction relies on the correlations between life history variables with body mass, brain size and dental development status, i.e. variables that can be obtained -directly or indirectly- from skeletal or fossilised remains. Traditionally, dental eruption is regarded as particularly informative, especially when combined with histological analyses that determine the absolute timing of events [3], [4]. Such approaches are rooted in the observation that first permanent molar eruption is correlated with adult brain size [5], [6] and, consequently, other life history variables [7], [8]. Yet, both brain development [9], [10] and molar eruption [11]–[13] vary considerably in response to resource availability and its effects on maternal (and infant) energetics [14], [15] and the pace of life in general [16]–[18]. Physiological constraints, behavioural factors and intraspecies competition may however obviate predictions made on the basis of energetics alone [19], [20]. Hence, the body of anthropological and neontological evidence cautions against an overemphasis of the absolute timing of dental development as a means to determine whether or not hominin life histories have advanced relative to the great ape condition [21]. Yet, the premise that some 92%–96% of adult brain size is achieved by the time first permanent molars erupt could still be valid [12], [22]. Such a correlation would conceivably result from differences in brain size being brought about by changes in growth rates during pre-natal and early post-natal development [9], [10], [23]–[26]. Alternatively, or in combination, both tooth eruption and brain development may respond to the same external stimuli and selection pressures. For instance, first permanent molar eruption is generally considered to mark the attainment of masticatory competency both with regard to morphology and motor-neuron control [27], as well as an infant’s ability to survive without its mother. For survival, an infant not only needs the appropriate physical attributes, but must also have acquired the necessary foraging skills and predator avoidance strategies [18], [28]. Synchronisation of physical and neurological development therefore seems logical and would be particularly important when the environment is resource-limited and/or non-density dependent mortality is high [1]. In the relative absence of such external constraints, it is conceivable that the association between dental eruption and brain development may become more relaxed. Analysis of an historical skeletal sample of great apes from Cameroon, West Africa, affords the opportunity to test this possibility while controlling for the effects of habitat and ecology.

West African lowland gorillas and chimpanzees inhabit primary and old secondary forests; both consume mainly fruits complemented by vegetative matter [29]–[33] and small quantities of invertebrates [34]–[36]. Despite their overlapping diets, lowland gorillas are more selective in the choice of fruits than chimpanzees, but are more flexible when choosing non-fruit vegetative matter, especially during times of seasonal or annual fruit scarcity [37]. Across sites, and between sexes, dietary variation appears surprisingly limited, however, and suggests that gorillas pursue a feeding strategy that maintains nutritional balance [38], [39]. The generally broader diet of gorillas can be understood as a consequence of the species’ larger body mass [40], [41], as predicted for sympatric species from the same level of the foodweb [42]. Chimpanzees respond differently to intra- and interannual resource limitations. Rather than adjusting their diet, they change their sociality, i.e. group size, thereby reducing intragroup competition [31].

The greater frugivory (and presumably greater energetic yield) of Western lowland gorillas, compared to mountain gorillas, affects the species’ ranging pattern [43], sociality and life histories [44], [45], as it does in chimpanzees. Available neontological data indicate that juvenile mortality is lower and interbirth intervals are longer in Western lowland gorillas, whilst overall physical maturation proceeds at a much slower pace [2], [46]. Specifically, weaning occurs some 16 months later than in mountain gorillas, such that suckling continues to a median age of 4 ½ years –4 ¾ years [46], [47]. The delayed developmental schedule of Western lowland gorillas is therefore more akin to that of chimpanzees [2], [48] than to mountain gorillas [49], [50]. Whether this delay affects all developmental systems equally is not clear however. Here we explore whether both dental development and brain growth follow the same pattern in sympatric *Pan troglodytes* and *Gorilla gorilla*, after having controlled for the effects of habitat, both physical and as exploited by the species. The latter was determined through the use of stable isotope analyses of hair from associated skins held at the museum. The combined morphological and biochemical approach allows us to address the following questions:

Do stable isotope ratios show distinct ecological niches for *Gorilla gorilla* and *Pan troglodytes* from Central Cameroon?
What are the effects of sympatry on habitat exploitation by both species?
Are there discernible effects of ecology on physical development, i.e. with regard to dental development and brain size?
What are the evolutionary implications of the findings?

## Materials and Methods

**Figure 1:**
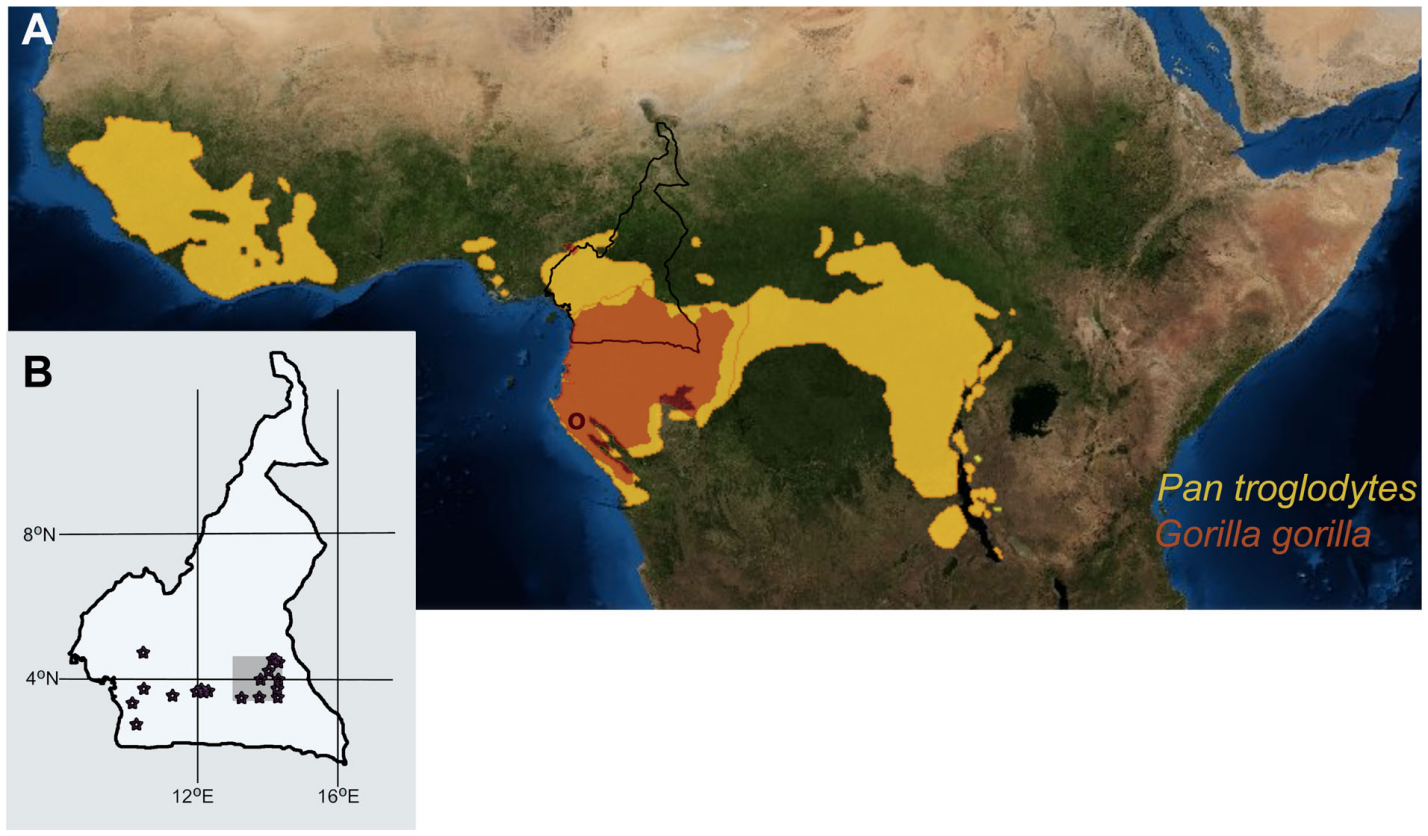
In (**A**) the distribution of *Gorilla gorilla* and *Pan troglodytes* across Central Africa is shown, modified from *The IUCN Red List of Threatened Species* (<http://www.iucnredlist.org>). Black circle indicates the location of the study site in Gabon [60]. In (**B**) the locations within the transect of Cameroon from where the samples are derived are shown by asterisks. Grey box outlines the skeletal material sampling area.

**Figure 2:**
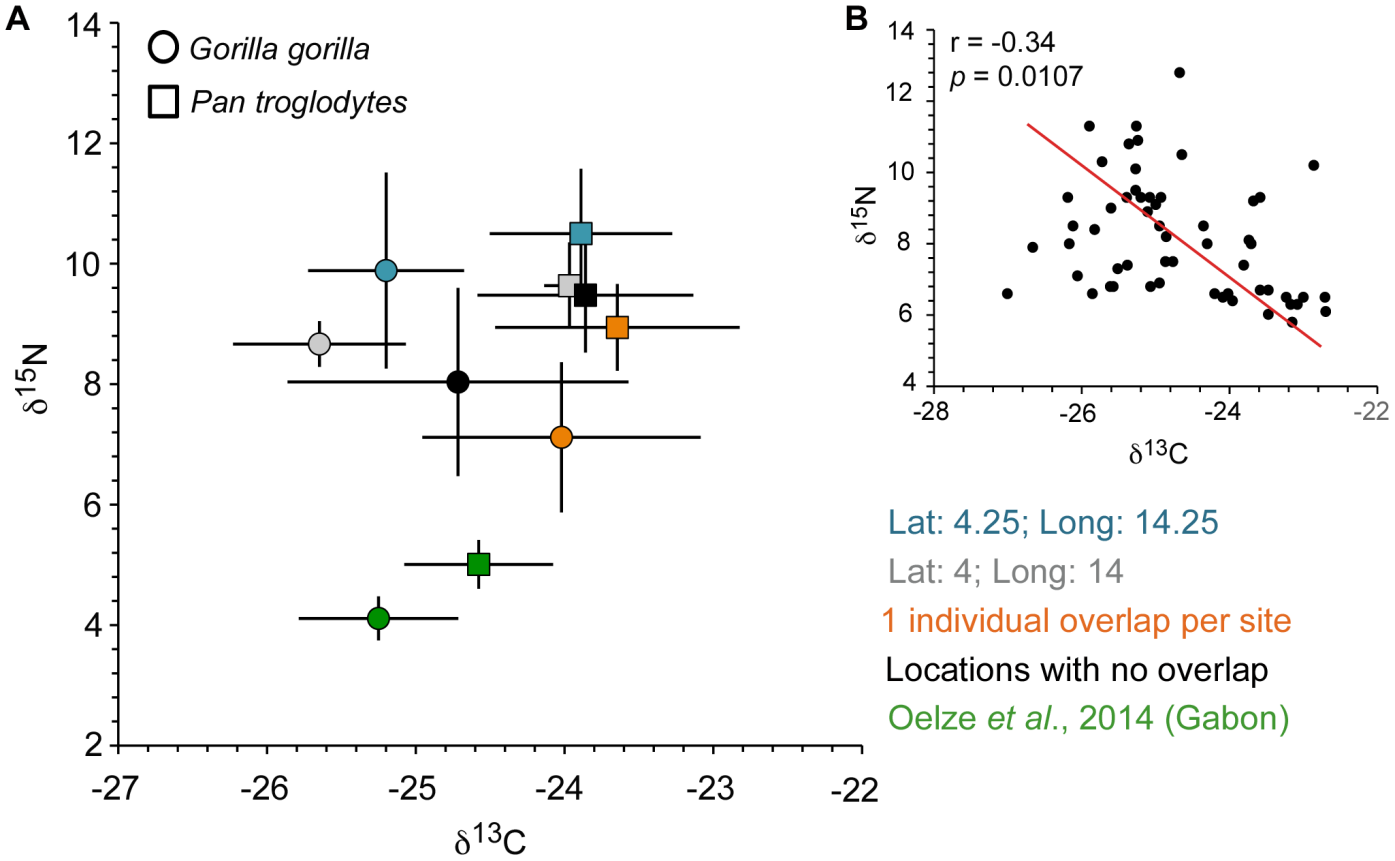
The stable isotope results for δ^13^C and δ^15^N obtained from primate hair are shown in (**A**). All hair isotope data are expressed in per mil (^o^/_oo_) relative to VPDB and AIR for d^13^C and δ^15^N, respectively. The sample is divided into locations from which both species were recorded (grey, blue), locations where there was only moderate overlap, i.e. one individual from one species and several from the other (orange) and locations that exclusively recorded either chimpanzees or gorillas (black). In comparison, the results given in [61] for sympatric gorillas and chimpanzees from Gabon are also indicated. In (**B**) the stable isotope data for all gorilla individuals are shown. The Reduced Major Axis line is shown (red) and the correlation and significance level is given.

The material used in this study was wild shot by Major Percy Powell-Cotton between April 1927 and June 1935 in the course of several expeditions, and is now housed at the Powell-Cotton Museum at Birchington, Kent (UK). A recent review details the history of the collection and provides a summary of the developmental status (eruption, sutures, epiphysial fusion) of the specimens collected [51]. Although geographically relatively restricted when compared to other major primate collections, the material at the Powell-Cotton Museum still emanates from a large geographic area. This is potentially problematic when aiming to discern interactions between habitat use, ecology and primate biology. As most of the specimens had the locations recorded at the time, and many preserve skins from which hair could be extracted, differences in habitat exploitation/dietary ecology could therefore be appraised independently prior to selection of skeletal material for developmental study. First, only material from a West-East transect of central Cameroon was considered for analyses (Figure 1; Table S1 in File S1). Second, within this relatively narrow strip of land differences in habitat exploitation by the great apes were identified through stable isotope analyses of hair (Figure 2). Third, in accordance with the availability of skeletal material, the study area was restricted further: skulls of sympatric *Gorilla gorilla* and *Pan troglodytes* from locations between 13°E–14.25°E longitude and 3.25°N–4.25°N latitude were selected for morphological analyses; this translates to 1268.5 km^2^.

Stable isotope analyses were performed on hair samples of 55 gorillas and 39 chimpanzees from a geographic transect of Central Cameroon (Figure 1, Table S1 in File S1). The skins in the Powell-Cotton museum collection have not been treated with chemical preservatives (Angela Gill, pers. comm.) and the hair is well-preserved and clean, lacking any residues. Individual hair samples removed for analyses were cleaned in purified water [52] and dried before being loaded into tin capsules for isotopic analysis. Single analyses of individual hairs were carried out for δ^13^C and δ^15^N. By convention the results are reported in the δ notation relative to the international standards VPDB for ^13^C/^12^C, and AIR for ^15^N/^14^N. The isotope data were normalised using four standards (USGS40, IAEA CH6, and internal laboratory standards Alanine and “Bovine collagen”). Precision as indicated by repetitive measurements of the Alanine standard is ±0.1^o^/_oo_, and ±0.3^o^/_oo_ for δ^13^C and δ^15^N, respectively. Patterns of difference in isotope composition within and between sites informed the selection of skulls for scanning (Figure 2).

In total, an ontogenetic series of 75 skulls (*Gorilla* male: 21; *Gorilla* female: 14; *Pan* male: 23; *Pan* female: 17) was available for study of dental developmental status and endocranial volume, 51 of which (*Gorilla*: 21; *Pan*: 30) also had associated skins (Table S1 in File SI). All material is recorded as being derived from locations between 13°E – 14.25°E longitude, and 3.25°N – 4.25°N latitude (Figure 1B, grey box). The skulls were scanned at the William Harvey Hospital, Kent (UK) with a Siemens Somatom Definition Flash CT scanner, at a slice thickness of 0.2 mm. Although every effort was made to scan all material from this restricted area, this was not possible. As the Powell-Cotton Museum does not have in-house scanning facilities, the number of skulls available for scanning needed to be limited, not least because only a few specimens can be transported for scanning at the hospital some 60 km away at any one time, in order to prevent potential damage or loss [51]. Endocranial volumes (ECV) in cc were determined in AMIRA (http://www.vsg3d.com/amira) following the protocol outlined in [53]. The data were validated through independent segmentation of 5 skulls, which found ECV to be consistently below a 1% error margin.

**Figure 3:**
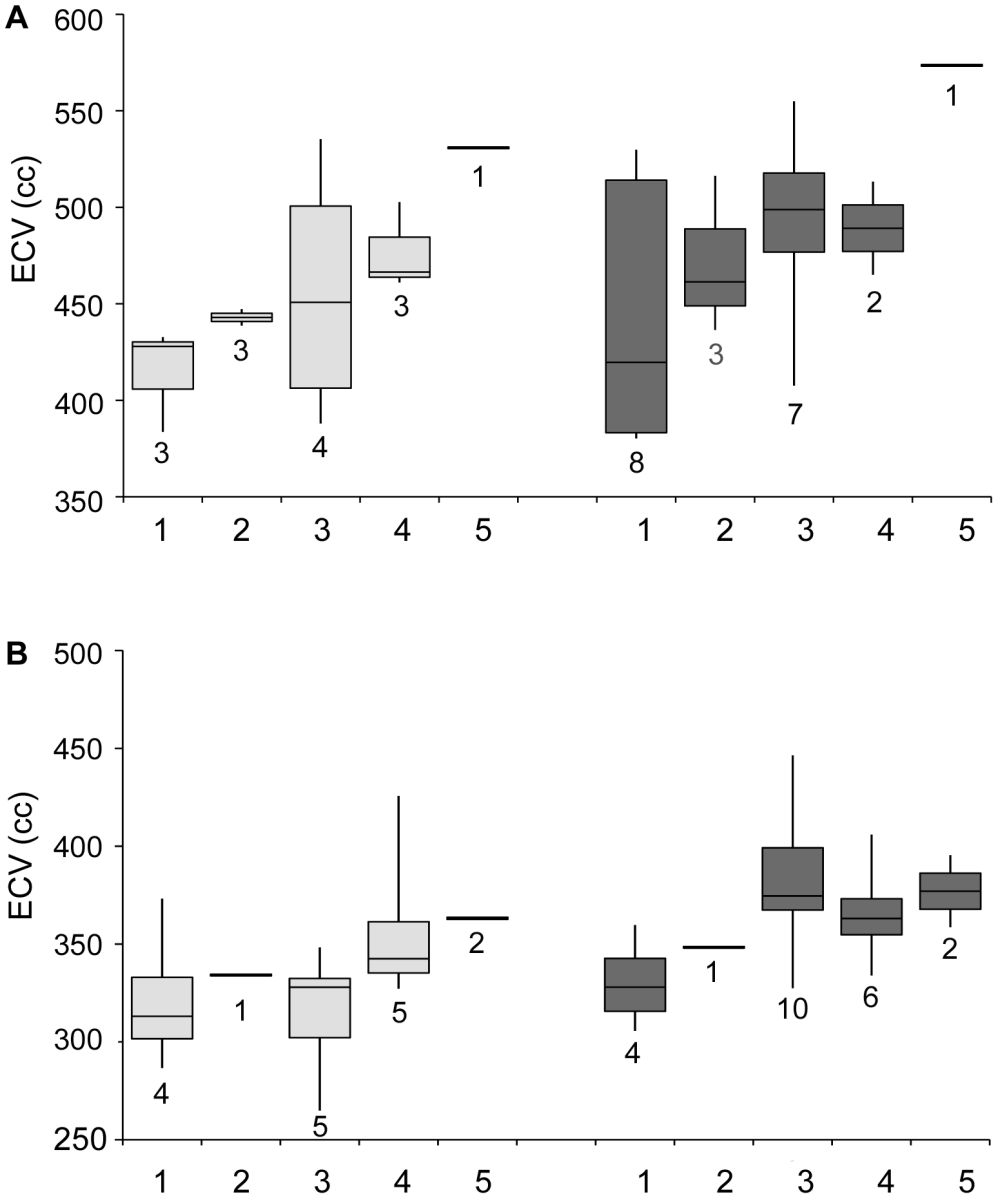
Boxplot of brain sizes by developmental stages for (A) gorillas and (B) chimpanzees. Females are shown in light grey and males in dark grey. Group 1: individuals with deciduous dentition only, Group 2: individuals with alveolar emergence of M1 up to M1 being in functional occlusion, Group 3: individuals with M1 in functional occlusion up to M2 being in functional occlusion, Group 4: M2 in occlusion to M3 reaching the occlusal plane, Group 5: adult. Sample sizes for each boxplot are indicated at each lower end, whilst descriptive statistics are given in Table S4 in File S1.

Mineralization of mandibular teeth was recorded following standard procedures [54] (Table S2 in File SI). Eruption status for each tooth was independently recorded in 5 stages (1 = emergence initiated, i.e. broken through the alveolar margin, 2 = half of the tooth crown above the alveolar margin, 3 = emerged, but not yet in functional occlusion, 4 = in functional occlusion, 5 = shed or lost). Both sets were arranged in order of ascending maturity [55] for further analyses. The distinction between mineralization and eruption may hold information with regard to the influence of environmental versus genetic factors: dental mineralization is generally considered to be a good estimator of species-specific development [56], [57], albeit with some degree of polymorphism [58], also observed in the present sample and compounding straight-forward seriation of specimens. In contrast, eruption of teeth is more susceptible to external influences, such as nutrition and hormones [59], [60]. Hence, although correlated, both measures of dental development provide slightly different information with regard to an individual’s overall maturity and, possibly, nutritional status during early postnatal development. Very young and adult specimens are underrepresented in the sample and the categorization for maturation used in [12] was therefore modified (Figure 3): Group 1 consists of individuals with deciduous dentition only, Group 2 includes individuals with alveolar emergence of M1 up to M1 being in functional occlusion, Group 3 contains individuals with M1 in functional occlusion up to M2 being in functional occlusion, Group 4 is from M2 in occlusion to M3 reaching the occlusal plane, Group 5 consists of fully adult specimens.

All statistical analyses were carried out in either Excel or the free downloadable programme *PAST* [61]. To test for the strength of relationships (or lack thereof) bootstrap analyses, i.e. resampling with replacement (n = 1000), were carried out on relevant subsamples.

## Results

The isotope data are shown in Figure 2 and compared to those published for sympatric great apes in Gabon [62]. Although mean δ^13^C and δ^15^N values differ across sites, the overall patterns are similar such that at any location gorillas tend to be more depleted in δ^13^C and to have slightly lower δ^15^N values when sympatric with chimpanzees (Figure 2A). The Powell-Cotton collection predates the major impact of fossil fuel burning on modern global CO_2_ δ^13^C values. The δ^13^C values in current primate studies, e.g., those reported recently for Gabon, the Congo and Cote d’Ivoire [62]–[65], would reflect this impact, on the order of −1.5^o^/_oo_ or more [66]. Consequently, the historical and modern datasets are not directly comparable unless all values are adjusted to pre-Industrial levels. We have made no adjustments here since, firstly, we are primarily interested in comparisons within the Powell-Cotton Cameroon collection and, secondly, because δ^13^C_CO2_ in 1930 differs from pre-industrial values by only ca. 0.2^o^/_oo_ [66]. It is worth pointing out however, that the δ^13^C values observed in the present study indicate moderately closed environments, whilst differences in δ^15^N between the Cameroon and Gabon studies are likely due to differences in soil nitrogen status and plant root specialisation in differing environments [67].

Species differences are less apparent when the entire Cameroon sample is considered, mostly due to the range of variation found in gorillas being considerably larger than that in chimpanzees (δ^13^C: *Gorilla*: mean = −24.69, std = 1.09, cv = −4.41; *Pan*: mean = −23.83, std = 0.67, cv = −2.79; Wilcoxon Mann-Whitney *U* = 601, *p* = 0.0003); δ^15^N: *Gorilla*: mean = 8.12, std = 1.65, cv = 20.25; *Pan*: mean = 9.37, std = 0.89, cv = 9.49; *U = *530, *p*<0.0001). In gorillas, δ^15^N decreases significantly as δ^13^C becomes more positive (*r* = −0.341, *p = *0.011) (Figure 2B), whereas no correlation was found in chimpanzees (*r* = −0.099, *p* = 0.5478). The overlap across the entire sample, on the one hand, and the marked distinctions at shared locations, on the other, suggests consistent differences in habitat exploitation at the local level, which could impact on a species’ biology. Analyses of skeletal material at the level of the species may therefore mask biologically relevant information (Figure 1, 2).

As regards the more restricted geographic area from which the skulls were selected to determine differences in developmental patterns (Figure 1B, grey box; Table S1 in File SI) elevations range from 659 m to 670 m, average annual rainfall is 1700 mm per year, and the vegetation is described as closed evergreen and semi-evergreen lowland forest with some deciduous elements [68]. Despite their overlapping environment, *Gorilla* and *Pan* differ significantly in δ^13^C (*Gorilla*: mean = −25.37, std = 0.47, cv = −1.86; *Pan*: mean = −23.93, std = 0.39, cv = 1.65) (Wilcoxon Mann-Whitney *U* = 4, *p*<0.0001). Conversely, no differences were found in δ^15^N (*Gorilla*: mean = 9.68, std = 1.23, cv = 12.74; *Pan*: mean = 9.52, std = 0.84, cv = 0.78; *U = *308, *p = *0.8982), although these means mask variability related to age, i.e. infant, juvenile and adult, in both species (see below). The low δ^13^C values are typical for primates in a tropical forest system [64], [65], but do not indicate foraging under the densest canopy, as indicated for instance by *Tragelaphus eurycerus* individuals from the same area that show δ^13^C values below −30^o^/_oo_ (Lee-Thorp and Macho, unpubl. data).

To assess whether the skeletal subsample selected here may indeed warrant separate analysis from the remainder of the Powell-Cotton Museum, adult brain sizes were first compared with those published in [69]; the latter study used traditional methods (i.e., filling the cranial cavity with mustard seeds) for the determination of ECVs. The material used in this earlier study derived to a large extent, but not exclusively, from the Powell-Cotton Museum. Specimen numbers are not listed and the extent of sample overlap can therefore not be fully assessed; only mean values and standard deviations are available for comparison (Figure S1 in File S1). The morphological variation yielded in [69] exceeds that calculated here, and the morphological variation for chimpanzees is consistently higher. Although the values obtained for gorillas in the present study center around the mean given in [69], sexual dimorphism is less marked, notably due to the larger average brain sizes of females. It is noteworthy that our means do not deviate systematically from those published by Ashton and Spence [69], e.g. being either consistently larger or smaller. A methodological bias therefore seems unlikely. Perhaps most importantly, bootstrapping, i.e. resampling with replacement (n = 1000), found the present sample to be statistically indistinguishable from the calculated population means (Table S3 in File S1). Furthermore, the age categories used by Ashton and colleagues [69], [70] are not well defined and, when approximated in line with the present analyses, show gorillas to exhibit greater maturity at each stage of development than found here (see below). In summary, although a sampling bias cannot be ruled out completely, the consistently lower variation found in the present analyses, the non-directional differences observed and the results obtained by resampling, suggest that subtle regional differences were missed in previous studies.

Spearman’s Rank-order correlations ρ between mineralization and eruption are significant at *P<*0.0001 (*r_Gorilla_*_-female = _0.98; *r_Gorilla_*_-male = _0.98; *r_Pan_*_-female_ *= *0.98; *r_Pan_*_-male = _0.97), whereby the association between eruption and mineralization is least tight between M1 and M2 eruption. This confirms suggestions that both measures reflect slightly different aspects of an animal’s development, although the differences (and sample sizes) found are too small to allow meaningful interpretation and/or more sophisticated statistical analyses [71]. Conversely, the correlation of either mineralization or eruption with ECV is relatively poor (mineralization: *r_Gorilla_*_-female = _0.56, *P*<0.05; *r_Gorilla_*_-male = _0.45, *P*<0.05; *r_Pan_*_-female_ *= *0.55, *P*<0.05; *r_Pan_*_-male = _0.50, n.s.; eruption: *r_Gorilla_*_-female = _0.58, *P*<0.05; *r_Gorilla_*_-male = _0.43, *P*<0.05; *r_Pan_*_-female_ *= *0.60, *P*<0.05; *r_Pan_*_-male = _0.57, *P*<0.05). There is a slight tendency for the correlations between eruption and ECV to be higher than between mineralization and ECV. Figure 3 shows the boxplot for ECV increase with eruption stages (Table S4 in File S1). As groups are not represented by equal numbers and, owing to small sample sizes, caution needs to be exercised when interpreting the trends. For example, there appears a trend towards a steady increase in ECV in *Gorilla* (ANOVA: *F_Gorilla_*_-female = _2.75, *P* = 0.089, *F_Gorilla_*_-male = _2.51, *P* = 0.080) but this is largely driven by the large adult specimens. In chimpanzees, an increase appears to occur in males (*F_Pan_*_-male = _2.72, *P* = 0.059), but not females (*F_Pan_*_-female = _1.68, *P* = 0.2104) (Figure 3, Table S4 in File S1).

**Figure 4:**
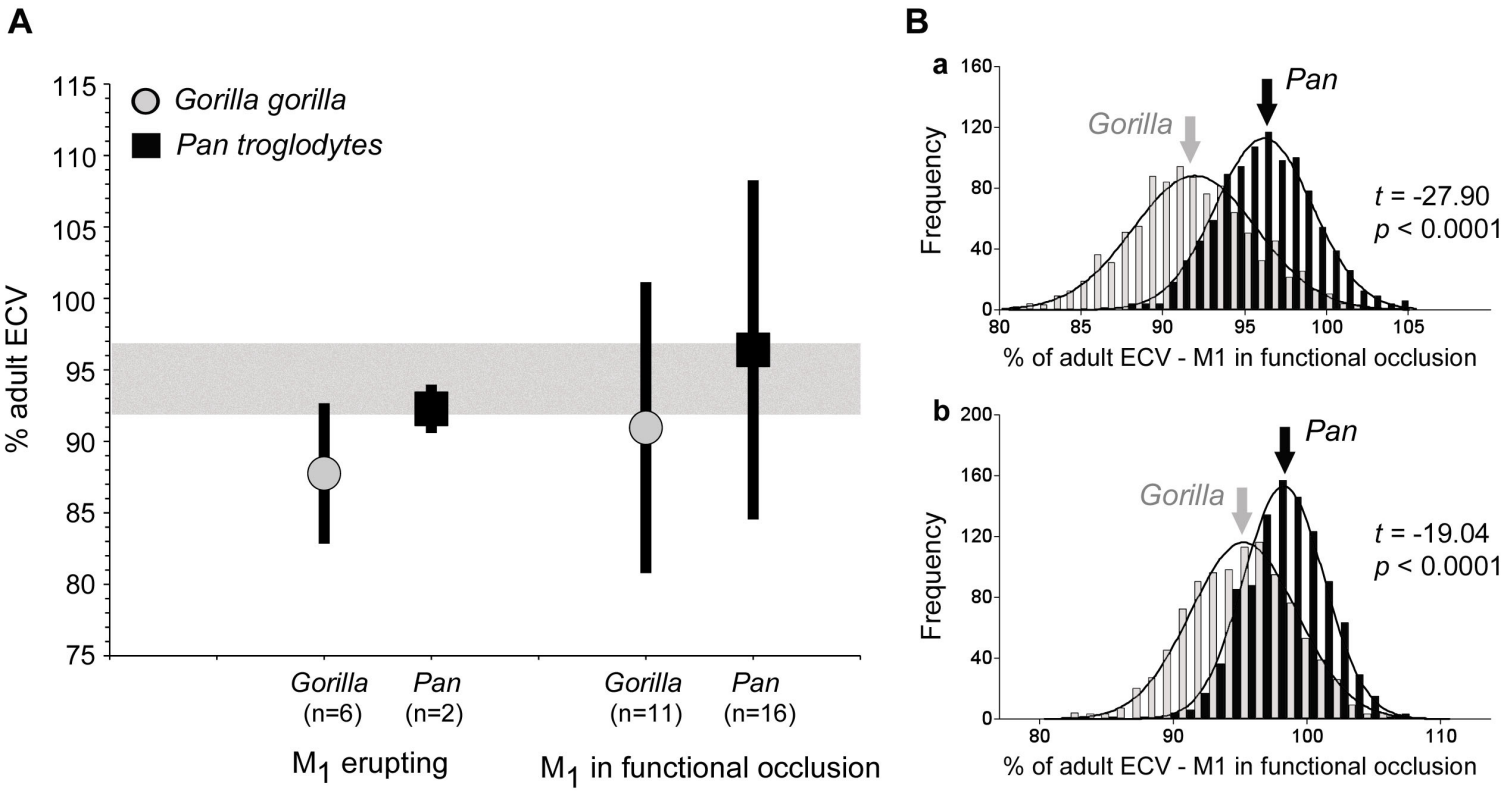
Percentage of adult brain size achieved by the time M1 starts to erupt (Group 2), and once they have come into functional occlusion until M2 eruption is complete (Group 3), are shown for gorillas and chimpanzees respectively (**A**). The bars represent mean values and standard deviations. To test whether there are differences in % ECV attained in Group 3 between gorillas and chimpanzees, the samples were bootstrapped (resampling with replacement) and statistically tested (**Ba**). In (**Bb**) all individuals with M2 erupted were considered adult for the calculation of adult brain size and the analyses were repeated. Despite smaller calculated adult brain sizes, the difference between *Gorilla* and *Pan* remained statistically significant at the 0.0001% probability level.

The average adult ECVs were then computed and used to determine the percentage of ECV attained by individuals in group 2 (alveolar emergence of M1 up to M1 being in full functional occlusion) and group 3 (M1 in occlusion until M2 is in full functional occlusion), in order to test whether some 92%−96% of adult brain size is indeed achieved by the time first permanent molars erupt [12], [22]. Sex differences were accounted for by calculating the percentage for each sex separately before pooling the results for analyses of species-specific differences (Figure 4). In order to reduce a sampling bias in gorilla (see above), two specimens with M3 nearly erupted were considered adult for this analysis (Table S1 in File S1). This reduced the average adult brain size for gorilla by some 11.5%; these latter figures are considered more representative of the population as a whole. Viewed as a percentage, gorilla brain sizes are consistently lower during infancy than would be expected on the basis of their dental developmental status and using chimpanzees as a yardstick: at the time M1 start to erupt, only 87.76% of adult ECV has been achieved, whereas it is 92.28% in chimpanzees. Sample sizes for this age category are particularly small however, and the possibility of a sampling error (Type I) cannot be ruled out. In contrast, when M1 are already in functional occlusion, sample sizes are more adequate and comparisons between species are more reliable. At this stage of development gorillas lag behind sympatric chimpanzees in brain size development also, the figures being 90.96% and 96.40%, respectively. A consistent developmental trend thus seems likely, although the results are not statistically significant (student’s *t = *−13.26, *p = *0.1991) (Figure 4A). To further test the strength (or lack) of this relationship, resampling with replacement (n = 1000) was carried out. No significant differences in sample means were found (Table S3 in File S1). Contrary to analyses of the raw data however, differences between species are highly significant after bootstrapping (Figure 4Ba). Even when all individuals with M2 in full occlusion are considered adult for the purpose of calculating adult brain size, thereby effectively lowering adult ECVs, gorillas maintain lower ECVs than chimpanzees at comparable developmental stages (Figure 4Bb). This correlates with, and may be a consequence of, the apparently delayed onset of adult feeding behaviour in this species.

**Figure 5:**
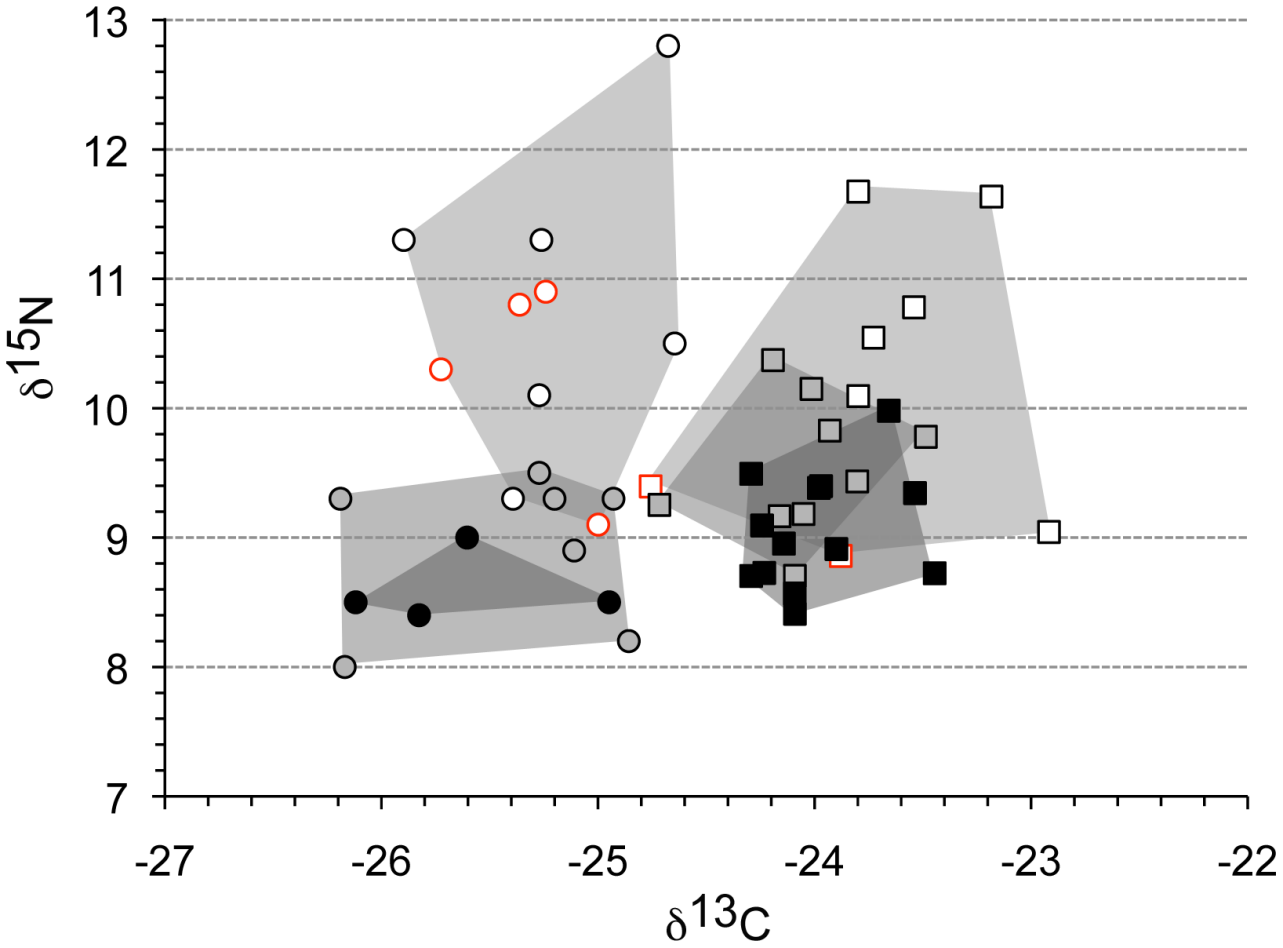
Stable isotope data for the subsample of *Gorilla* (○) and *Pan* (□) hair (see grey box in Figure 1B) that also preserve skulls from the area used for analyses of morphological/developmental differences. Separation in isotope space between infants (light grey), i.e. individuals prior to M1 being in functional occlusion, juveniles (middle grey) and adults (dark grey), i.e. after M2 in functional occlusion (dark grey) are also shown. Individuals with M1 in the process of eruption are indicated in red.

The δ^15^N values for gorillas in group 2 (i.e., individuals with M1 erupting) are about 10.3^o^/_oo_ as opposed to 8.9^o^/_oo_ in the subsequent developmental group when M1s are in functional occlusion (Wilcoxon Mann-Whitney *U* = 4, *p* = 0.07). The corresponding values for chimpanzees are ca. 9.1^o^/_oo_ and 9.5^o^/_oo_, respectively (*U* = 5, *p* = 0.4094). No statistical differences in δ^13^C were observed between these developmental stages (*Gorilla*: *U* = 0.12, p = 0.7768; *Pan*: *U* = 7, *p* = 0.7237). Perhaps most surprisingly, 3 of the 4 gorilla individuals with erupting M1 fall well within the range of infant specimens as regards the δ^15^N of their hair (Figure 5, red circles), whereas chimpanzees at this developmental stage are within the range of adults (Figure 5, red squares). This is not a function of sex as males and females are distributed equally in this age group in gorillas and chimpanzees. Overall, δ^15^N differences between developmental stages are more marked in gorillas (ANOVA: *F* = 12.76, *p* = 0.00035) than in chimpanzees (ANOVA: *F* = 7.38, *p* = 0.0028) (Figure 5, Figure S2 in File S1). There are no statistically significant differences in δ^13^C (ANOVA: *Gorilla*: *F* = 0. 92, *p* = 0.4177; *Pan*: *F* = 2.046, *p* = 0.1488), although there appears a slight trend for chimpanzee hair to become more depleted in δ^13^C as the individuals mature (Figure 5, Figure S2 in File S1).

## Discussion

Behavioural data on West African great apes are rare and insights into gorilla life histories have become available only fairly recently [2], [45]–[47]; information about their skeletal development is extremely limited too [72]. Yet, such information is essential for conservation efforts, for a better understanding of developmental plasticity against ecological conditions [73] and for an appraisal of life history evolution in extinct hominins. The interplay between resource availability, habitat type and competitive exclusion is complex however [74], making it difficult to isolate correlation from causation. In this study we assessed whether information pertaining to these issues can be extracted from a historical collection, using a combination of morphological and isotopic approaches.

The present material is derived from a 20^th^ century museum collection and, when the sample is divided into smaller, e.g. geographical, units, sample sizes inevitably become small. Nonetheless, the extent and patterning of habitat partitioning found in the present study has a bearing on our understanding of the behavioural ecology of sympatric apes and on great ape physiology. When combined with morphological data, insights into the relationship between ecology and development emerge, which have implications for an interpretation of life history evolution among hominoids including hominins.

As anticipated on the basis of similarities in diet, gorillas and chimpanzees overlap in isotope values when the samples are considered across the entire transect in Cameroon. The lower variation in isotope values in chimpanzees is consistent with the greater fidelity in food choice of this species (Figure 2), while the broader variation in gorillas may reflect their ability to adjust their feeding strategy and use of habitat. When gorillas and chimpanzees share the same physical habitat however, the separation in carbon isotope space is marked, even complete (Figure 5). Lower δ^13^C values for gorillas, and conversely higher values for chimpanzees, is a strong indication of niche partitioning in this habitat. It must be related to a differential emphasis on particular forest resources, in particular differences in the plant taxa and growth forms typically consumed by gorillas and chimpanzees, and where they are located in the forest ecosystem. For instance, in the Gabon Loango National Park study some of the taxa consumed by gorillas alone are depleted in ^13^C [62], while chimpanzees’ greater reliance on frugivory may reflect in some measure the typically slightly higher δ^13^C values of fruit as a non-photosynthetic tissue compared to leaves [75]. In Loango, the mean δ^13^C difference between sympatric gorillas and chimpanzees is smaller (0.7^ o^/_oo_) than it is in the Cameroon sample (1.5^ o^/_oo_). Interpretation of this difference in terms of frugivory versus folivory alone requires minimal fruit and maximum δ^13^C-depleted leaves in gorilla diets, which fits ill with observational data [37]. Given the well-known canopy effect in tropical forests [76], in which recycling of respired CO_2_ and low light under a dense tree canopy combine to produce low δ^13^C values in plants, we argue that the present data indicate also exploitation of plants under denser canopy conditions by gorillas than by chimpanzees. This argument is consistent with behavioural studies and highlights the importance of competition in niche separation [31]–[33], [36], [77].

The systematic pattern of lower δ^15^N for gorillas at each location over the entire geographic area sampled is perhaps more difficult to interpret (Figure 2). One explanation is that in each location gorillas simply consumed more plants with lower δ^15^N composition than did chimpanzees. However, no overall δ^15^N differences were found in plants eaten by gorillas and chimpanzees in Loango [62]. Higher δ^15^N values for chimpanzees at each location could simply reflect the consumption of slightly more invertebrate or vertebrate animal foods [78]. The mean δ^15^N differences are quite small however and differences in digestive morphology and physiology, microbiome [79] and gut retention time [80] likely also influenced the magnitude of nitrogen-isotope fractionation between diet and tissues in the two taxa. These possibilities are difficult to evaluate on present evidence, also because they likely act in combination rather than isolation.

Regardless, and important for an assessment of gorilla life history, there is no evidence in the literature that gorilla diets are low in protein, or nutritional value generally, compared to chimpanzee diets, which could account for their delayed development (Figure 4). As a case in point, Rothman and colleagues [81] argued that the amount of protein eaten by mountain gorillas exceeds their daily requirements even after protein intake was corrected for the effects of condensed tannins [82] and the fiber-bound unavailable N [83], [84]; the high-protein diet and its consistent availability underlies the high growth rates observed in mountain gorillas [19]. Western lowland gorillas, too, avoid foods rich in tannins and lignin [39], which could potentially offset their greater consumption of protein-rich vegetative matter. Perhaps more informative for the present purpose therefore are reports of negative energy balance, including weight loss during periods of food scarcity, in chimpanzees [85]–[88], but not in gorillas. Also, 50% of chimpanzees from Mahale National Park, Tanzania, were found to have elevated levels of protein in their urine, even though they showed no obvious health problems [89]. For chimpanzees at Kibale National Park, Uganda, 29% of chimpanzees had moderate levels of protein in their urine, and 33% showed severe levels. The figures for Western lowland gorillas from Bai Hokou, Central African Republic, are 3% (moderate) and 0% (severe), respectively [90]; gorillas, especially juveniles, seem more affected by parasite infections during seasonal resource fluctuations [90].

On the basis of the evidence presently available, it is parsimonious to suggest that gorillas do not suffer from nutritional stress and protein deficiency. The lower δ^15^N values in gorillas across the range most probably reflect subtle differences in plant food isotope composition and physiology and little, if any, animal food, but not lack of protein. A calorie- and protein-impoverished habitat/diet is thus unlikely to have compromised the reproductive success of the gorillas studied here. Instead, habitat exploitation (rather than nutritional deficiency) is suggested to primarily account for the differences in developmental patterns found between the sympatric great apes (Figures 3–5).

Despite modest sample sizes, an uneven distribution of individuals in each age class, and a lack of a representative number of infants, where most of brain growth is expected to occur [10], [12], [23], [25], gorillas consistently show brain development that is delayed with respect to dental eruption (or dental development accelerated with regard to brain size): only some 88% of adult brain size has been achieved by the time M1 erupt and 91% when it is already in functional occlusion. This renders molar eruption, like molar crown formation [91], a poor predictor for brain development in extinct hominins [92] despite high correlations across anthropoids [5], [93]. Because of the relatively short period during which the material was sampled, i.e. 8 years, the results are not likely artefacts due to secular trends either [12]. Instead, ecological factors are implicated.

Frugivorous primates tend to have extended developmental schedules [9], [11], presumably to spread the risk of density dependent mortality that would occur from intra-specific competition for unevenly distributed seasonal food sources, i.e. the “risk aversion hypothesis” [19]. This contrasts with folivorous (and provisioned) primates, and helps to explain the fast life histories of mountain gorillas with regard to both dental development and brain growth cessation [12]; for *G. beringei* food sources are abundant, protein intake is high and competition low. Given the more strongly frugivorous nature of Western lowland gorilla diet [30]–[33] slower maturation rates are therefore expected and, indeed, have been observed [2], [45]–[47]. Although the present study cannot assess the absolute timing of developmental events and, hence, test the “risk aversion hypothesis” [19] directly, the overall habitat structure of the Cameroonian sites makes it parsimonious to infer that the gorillas studied here would follow the general, i.e. delayed, developmental schedule of other *G. gorilla* rather than the fast schedules of *G. beringei,* even if they consumed proportionally more (protein-rich) vegetative matter than sympatric chimpanzees (Figures 2, 5). The ***relative*** speed of developmental schedules, i.e. brain size development *versus* dental mineralization/eruption, on the other hand, can be determined. Which of the structures is delayed/accelerated with regard to the other is irrelevant however, as the biological outcome is the same, i.e. a dissociation of maturation states with potential consequences for fitness and survival of the animal.

Partitioning relates not only to the species’ dietary ecology [31]–[33], [62], [94], but also to physical aspects of the habitat (i.e., microhabitat) chosen for foraging (and other activities) by the great apes [95]. The combined effects of diet and habitat selection lessen inter-specific competition for resources, thereby improving the opportunities for both gorillas and chimpanzees to maintain nutrient-rich diets [29], [30]. For example, gorillas are more terrestrial and larger than chimpanzees and will therefore exploit different food sources. The low δ^13^C data for gorillas obtained here indicate that these animals foraged under denser forest canopy than chimpanzees. As argued above, it is implausible to attribute the average δ^13^C distinction of about 1.5^o^/_oo_ between sympatric gorillas and chimpanzees as due to differences in frugivory and folivory alone, as this would imply an almost complete reliance on fruit for chimpanzees and on vegetative matter for gorillas. Such a dietary division would render Western lowland gorillas more specialized than the folivorous mountain gorillas that consume fruits whenever the opportunity arises [81], and would conflict with behavioural evidence from Western Africa [37]. A preference for foraging in denser habitats must therefore contribute. This preference has consequences for predation risk [96].

Juveniles face greater risks of extrinsic mortality through predation than adults, which affects life history evolution [1]. For primates, Ross [97]–[99] showed that closed habitats apparently select for slow reproductive rates, even after the effects of body mass and environmental factors, like rainfall, have been taken into account. The denser forests occupied by gorillas are predicted to provide greater protection, especially from predation by leopards [96]. Because of their greater terrestriality gorillas are at a greater risk of predation, although their larger body masses and group cohesion offer some protection. Overall, gorilla infants/juveniles are generally better protected from extrinsic mortality than chimpanzees, save for infanticide [45]. Hence, juveniles of Western lowland gorillas can afford to delay (or dissociate) brain growth rates until after they have reached masticatory competency. The advantages are clear. Brain growth requires substantial amounts of energy [100] and extending, i.e. spreading, the growth period lowers the risk of starvation, especially when resources, such as fruits, are limited on a seasonal and/or interannual basis. Chimpanzees, in contrast, occupy more open forests (Figure 5) and, when resources become scarce, they reduce group size and disperse [101] rather than switch to alternative resources like gorillas. This strategy increases their exposure to predators. Juvenile chimpanzees should therefore have acquired the necessary neurological maturation, as well as masticatory competency, by the time they are weaned.

Differences in δ^15^N for infant, juvenile and adult gorillas are consistent with a scenario for prolonged nursing and dependency (and/or physiological immaturity), indicated by the elevation of δ^15^N in infants during nursing [102]–[104]. Figure 5 shows a gradual transition from higher to lower δ^15^N from infancy to adulthood, which in gorillas appears to be more abrupt, but also later during somatic development compared to chimpanzees. The combined evidence is consistent with propositions that chimpanzees are weaned (or have physiologically adjusted to an adult diet) by the time M1 come into functional occlusion whereas this process is delayed in gorillas, and is supported by observational data [46]. The energetic demands on the mother would be considerable however, as lactation is costly. A delay in weaning can therefore only be implemented when resources are sufficient and the maternal energy requirements are met while, at the same time, infant mortality rates remain low [17], [18]. Such conditions prevail in areas where plant biodiversity is high and predation low, i.e. in denser primary forests.

The outcomes of this study clearly add to the growing body of evidence that suggests that developmental schedules are variable and respond to environmental conditions [2], [9], [11], [20]. For palaeoanthropological purposes, the results caution against using M1 eruption (either absolutely or relatively) as a proxy for brain size and/or other life history variables. Where the reconstruction of early hominin life histories is concerned, this probably matters little. Hominins evolved in an increasingly open habitat [105], where predation risk was high [106]. A correlation between maturation schedules, i.e. morphological and neurological development, therefore seems likely [28]. However, once allocare became established [107] the synchronisation between developmental processes could have become relaxed. Hard evidence for such a proposition is not available to date, and may be difficult to find. Nonetheless, such a possibility needs to be entertained, not least because physiological data for modern humans suggest developmental plasticity to have played a key role in the evolution of hominin life histories [108]. Given the environmental fluctuations throughout hominin evolution, this seems intuitive. Based on the results of the present study, it now seems easily achievable also. Our closest relatives, the great apes, exhibit more diverse developmental schedules than previously assumed, apparently in response to different environmental conditions. Dietary flexibility and the ability to adjust to local situations appear at the heart of this developmental plasticity.

## Supporting Information

File S1 **Supporting Information.** Table S1 Primary data used. Table S2 Mineralization stages employed to assess the maturity of the specimens. Figure S1 Comparison of the present data with those published previously [65]. Table S3 Results of resampling procedures. Table S4 Descriptive statistics for endocranial volumes, by species and sex. Figure S2 Results of resampling procedure for δ^15^N and δ^13^C between infants, juveniles and adolescents/adults.
(PDF)
