# Supplementary material for: Niche Partitioning in Sympatric Gorilla and Pan from Cameroon: Implications for Life History Strategies and for Reconstructing the Evolution of Hominin Life History
Source: PLoS One. 2014 Jul 23;9(7):e102794. doi: 10.1371/journal.pone.0102794 (PMC4108342; doi:10.1371/journal.pone.0102794)
Supplement: File S1 — Supporting Information. Table S1 Primary data used. Table S2 Mineralization stages employed to assess the maturity of the specimens. Figure S1 Comparison of the present data with those published previously [65]. Table S3 Results of resampling procedures. Table S4 Descriptive statistics for endocranial volumes, by species and sex. Figure S2 Results of resampling procedure for δ15N and δ13C between infants, juveniles and adolescents/adults. (PDF) [file pone.0102794.s001.pdf]

Supporting Information

Macho GA, Lee-Thorp, JA: *Niche partitioning in sympatric Gorilla and Pan from Cameroon with possible effects on life history strategies: implications for reconstructing hominin life history evolution.*

Table S1 Specimens and primary data used for the present study, for 49 *Pan troglodytes* (N_{males} = 27, $N_{females}$ = 22) and 69 *Gorilla gorilla* (N_{males} = 37, $N_{females}$ = 32) . Only eruption status for permanent molars is shown (0 = mineralization not initiated; 1 = within the crypt; 2 = emergence initiated, cusp(s) above the aleveolar margin; 3 = emerged, but not yet in functional occlusion; 4 = functional occlusion; 5 = lost). Molar C/N ratios are also shown and indicate that all specimens, except M182 (*Pan*), have values regarded acceptable, i.e. between 3.4 and 4.1 [1].

**Species**	**No.**	**Lat. (N)**	**Long. (E)**	**Sex**	**ECV (cc)**	**Molar eruption status**			**C/N**	**$\delta^{15}N$**	**$\delta^{13}C$**
						**M1**	**M2**	**M3**			
Pan troglodytes											
	M276	4.5	10.5	F					3.6	8.0	-23.5
	CAMI.118	3.45	12.15	M					3.9	7.1	-21.5
	CAMI.222	3.45	12.3	M					3.8	9.3	-21.8
	M777	3.25	14.25	M					3.8	9.5	-24.1
	M300	3.75	14.25	F					3.7	8.7	-25.2
	M403	3.75	14.25	M					3.9	9.9	-23.7
	M451	3.75	14.25	F					3.6	8.3	-23.8
	M911	3.75	14.25	F					3.7	9.1	-23.8
	M275	3.75	14.25	F					4.0	10.1	-24.2
	CAMII.314	4.3	14.15	M	305.65	1	0	0	3.7	9.0	-22.9
	M105	3.75	13.75	F	362.84	4	4	4	3.7	8.6	-24.1
	M133 1st	4.1	14.1	F	332.43	4	1	0			
	M145	3.75	13.75	F	327.21	4	4	1	3.7	9.4	-24.0
	M152	3.75	13.25	M	337.00	1	0	0			
	M170	4.25	14.25	M	405.92	4	4	1	3.8	9.5	-24.3
	M173	4.25	14.25	M	359.71	1	1	0	3.9	11.6	-23.2
	M182	3.5	14.25	M	348.72	3/4	1	0	4.6	9.4	-24.8
	M250	3.25	14.25	M	365.68	4	1	1	3.6	9.2	-24.2
	M259	4	14	M	417.53	4	3	1	3.8	10.1	-24.0
	M274	3.25	14.25	F	342.51	4	4	1	3.7	10.0	-23.7
	M275	3.75	14.25	F	361.40	4	4	3			

	M300	3.75	14.25	F	302.13	4	1	1			
	M347	4	14	M	395.42	4	4	4			
	M358	4	14	M	327.44	4	2	1	3.7	9.8	-23.9
	M363	4	14	M	354.43	4	4	1	2.8	8.7	-24.2
	M369	4	14	M	348.14	4	1	1	3.9	8.7	-24.1
	M382	4	14	F	425.67	4	4	1	3.7	9.4	-24.0
	M403	3.75	14.25	M	382.00	4	1	0			
	M451	3.75	14.25	F	348.24	4	1	1			
	M453	3.25	14.25	M	358.59	4	4	4	3.7	9.1	-24.2
	M454	3.25	14.25	F	335.24	4	4	1	3.7	8.4	-24.1
	M455	3.25	14.25	M	374.02	4	4	1	3.6	9.3	-23.5
	M465	3.75	14.25	F	286.66	1	0	0	3.8	10.8	-23.5
	M475 2nd	3.75	14.25	F	306.61	1	1	0	4.0	11.7	-23.8
	M475A 1st	4	14	M	318.99	1	0	0	3.8	10.1	-23.8
	M506	3.75	14.25	F	363.99	4	4	4	3.7	8.7	-24.3
	M507	3.5	14.25	F	327.95	4	1	1	3.7	9.4	-23.8
	M52	3.75	13.75	M	355.68	4	4	1	3.7	8.7	-23.4
	M556	4	14	F	373.22	1	1	0	3.8	10.5	-23.7
	M60	3.75	13.75	M	373.46	4	2	1	3.9	9.3	-24.7
	M635	3.25	14.25	M	370.49	4	4	1	3.8	8.9	-23.9
	M636	3.25	14.25	M	333.98	4	4	1	3.9	9.0	-24.1
	M674	3.25	14.25	F	334.55	3/4	1	0	3.7	8.9	-23.9
	M744	4.25	14.25	M	404.93	4	1	1			
	M781	4.25	14.25	F	319.59	1	0	0			
	M876	4.25	14.25	M	375.69	4	1	1	4.0	10.4	-24.2
	M911	3.75	14.25	F	264.91	4	1	0			
	M93	3.75	13.75	M	446.42	4	1	1	3.8	9.8	-23.5
	M94	3.75	13.75	M	372.57	4	3	1	3.9	9.2	-24.1
Gorilla gorilla											
	ZII.64	3.1	10.2	M					3.7	6.8	-25.6
	CAMI.43	2.5	10.3	F					3.7	9.2	-23.7
	CAMI.41	2.5	10.3	M					3.5	6.6	-27.0
	CAMI.42	2.5	10.3	F					3.7	7.5	-24.9
	CAMI.44	2.5	10.3	F					3.7	7.4	-25.4
	CAMI.45	2.5	10.3	M					3.5	8.0	-23.7
	CAMI.46	2.5	10.3	M					3.7	6.8	-25.6
	CAMI.47	2.5	10.3	M					3.6	7.3	-25.5
	CAMI.48	2.5	10.3	M					3.6	8.0	-24.3
	CAMI.49	2.5	10.3	M					3.8	7.5	-24.8
	CAMI.50	2.5	10.3	F					3.5	7.1	-26.1
	CAMI.73	2.5	10.3	F					3.7	8.1	-23.7
	CAMI.14	3.5	10.5	F					3.6	7.9	-26.7
	CAMI.149	3.3	11.3	F					3.6	8.5	-24.4
	CAMI.150	3.3	11.3	F					3.7	9.3	-23.6
	CAMI.139	3.4	12	F					3.6	7.4	-23.8

CAMI.111	3.4	12.15	F					4.1	6.5	-23.0	
CAMI.106	3.4	12.15	M					3.6	6.6	-24.2	
CAMI.108	3.4	12.15	M					3.8	6.1	-22.7	
CAMI.109	3.4	12.15	F					3.7	6.3	-23.2	
CAMI.110	3.4	12.15	F					3.7	6.5	-22.7	
CAMI.208	3.4	12.15	M					3.6	5.8	-23.2	
CAMI.224	3.45	12.15	M					3.8	10.2	-22.9	
CAMI.95	3.45	12.15	F					3.6	6.6	-24.0	
CAMI.96	3.45	12.15	F					3.6	6.5	-24.1	
CAMI.97	3.45	12.15	F					3.9	6.7	-23.5	
CAMI.98	3.45	12.15	F					3.6	6.4	-24.0	
CAMI.99	3.45	12.15	M					3.6	6.7	-23.6	
CAMI.105	3.45	12.15	M					3.7	6.3	-23.1	
CAMI.134	3.45	12.15	M					3.6	6.5	-23.2	
CAMI.107	3.4	12.18	M					3.9	6.0	-23.5	
CAMII.323	4.3	14.15	M					3.5	6.6	-25.9	
CAMII.325	4.3	14.15	F					3.6	6.9	-24.9	
CAMII.324	4.3	14.18	F					3.6	6.8	-25.1	
CAMII 351	4.3	14.15	M	511.23	1	1	0				
M000	4.25	14.25	M	436.49	3	1	0	3.7	10.8	-25.4	
M109	4.25	14.25	M	513.26	4	4	2	3.6	8.5	-24.9	#
M117	3.25	13.25	F	432.68	1	1	0	3.6	10.1	-25.3	
M119 1st	3.25	13.25	M	554.92	4	1	0	3.8	8.2	-24.9	
M137	3.25	13.25	M	522.45	1	0	0	3.6	11.3	-25.9	
M141	3.25	13.25	M	443.37	1	1	0	3.6	11.3	-25.3	
M160	3.25	13.75	F	388.04	4	2	1	3.6	9.3	-24.9	
M169	3.25	13.75	M	496.65	4	1	1	3.7	9.3	-26.2	
M177	3.75	13.75	F	531.24	4	4	4				
M180 1st	4	14	F	502.72	4	4	1	3.6	8.4	-25.8	
M22	3.25	13.25	M	465.04	4	4	1				
M241		14.25	M	573.94	4	4	4				
M29	3.25	13.45	F	438.76	3	1	0	3.5	10.3	-25.7	
M319	4	14	F	535.27	4	1	0				
M32	3.25	13.25	M	396.03	1	1	0				
M33	3.25	13.45	M	505.07	4	1	1				
M333	4	14	F	442.96	2	1	0	3.6	9.1	-25.0	
M34	3.25	13.25	M	498.78	4	1	1	3.7	9.5	-25.3	
M35	3.25	13.25	F	466.45	4	4	3	3.7	9.0	-25.6	#
M387	4	14	F	461.07	4	4	1	4.1	8.5	-26.1	
M409	3.25	14.25	F	447.21	2	1	0				
M450	4	14	M	407.57	4	1	1				
M463	4.25	14.25	M	530.48	4	1	1	3.6	9.3	-25.2	
M471	4.25	14.25	M	516.24	3	1	1				
M476	4	14	M	383.23	1	0	0				
M487	4.25	14.25	M	456.92	4	1	0	3.8	8.0	-26.2	
M497	4.25	14.25	M	380.30	1	1	0				
M532	4.25	14.25	F	427.88	1	1	0	3.6	9.3	-25.4	
M625	3.25	14.25	F	489.11	4	1	1	3.7	8.9	-25.1	

M690 1st	4.25	14.25	M	529.81	1	1	0			
M756	4.25	14.25	M	383.21	1	0	0	3.6	10.5	-24.6
M887	4.25	14.25	F	383.72	1	0	0	3.6	12.8	-24.7
M98	3.25	13.25	F	412.36	4	1	0			
M99	3.25	13.25	M	461.35	3	1	0	3.7	10.9	-25.2

regarded adult for the purpose of calculating adult brain size (see main text).

Table S2 Tooth developmental stages for mandibular teeth, after Coquerelle et al. [2].

Stage	Description
0	Crypt formed but germ not visible.
a/A	Beginning of crown mineralization
b/B	Incisors and canine: Mineralized incisal edge/cusp tip has reached maximum mesiodistal width.<br>Molars: Coalescence of cusp tips to form a regularly outlined occlusal surface.
c/C	Enamel formation is complete at the occlusal surface. Approximal edges of forming crown have reached future contact areas. The beginning of a dentinal deposit is seen.
d/D	Crown formation is completed down to the cemento-enamel junction, at least in lateral projection (i.e., at mesial and distal faces of the tooth)
e/E	Incisors, canine, molars: Root formation is more than a spicule, but root length is less than crown height.<br>Molars: Initial formation of root bifurcation is seen in the form of a mineralized point or semilunar shape.<br>Root length is less than crown height.
f/F	Incisors, canine, molars: Root walls are very thin, and root length is equal to or greater than crown height. Root length is incomplete, with diverging apical edges.<br>Molars: Midway down root, root wall is thinner than root canal.
g/G	Incisors and canine: Root length is almost complete, but apical edges are parallel or slightly converging.<br>Molars: Mesial root length is almost complete, but apical edges are parallel or slightly converging. Midway down root, root wall is thicker than root canal.<br>Root length complete, with apical walls converging, but apex is still open (width = 1 mm).
h/H	Apical dentine edge is sharp; apex is only just visible/closed (width <1 mm).
r	Root resorption initiated.
x	Deciduous tooth exfoliated.

Species	No.	i1	i2	dc	dm1	dm2	I1	I2	C	P1	P2	M1	M2	M3
Pan troglodytes														
	CAMII.314	g	g	d	g	f	C	B	B	0 empty	0	C	0	0
	M105	x	x	x	x	x	H	H	H	H	H	H	H	H
	M133 1st	res	res	h	res	res	E	D	C	E	E	F	D	0
	M145	x	x	h	res	res	F erupted	F erupted	E	F	F	H	F erupted	C
	M152	g	g	e erupting	g	f erupted /g	C	C	B	0	0	C	0	0
	M170	x	x	res	x	x	H	H	E	H	G	H	G	A / Right Agenesis?
	M173	res	res	g	res	res	C	C	C	C	B	E	B	0
	M182	res	res by I1	res by I2	res	res	D	D	C	C	D	F erupting	C	0
	M250	res	h	h	res	res	D	C	C	C	D	F erupted	D	0
	M259	res	res by I1	h	res	res	E	E	C	E	E	H	E erupting	C
	M274	x	x	res by I2	x	x	F erupted	E erupting	D	E erupting	F erupting/ advance to P1	H	F erupted	C
	M275	x	x	x	x	x	H	H	G erupted	H	H	H	H	F erupting?
	M300	res	res by I1	h	res	res	E	E	C	E	E	G	E	B
	M347	res	res	res	res	res	H	H	F erupted	H	H	H	H	G limit H
	M358	x	res by I1	h	res	res	E	E	C	F	F	H	E	C
	M363	x	res by I2	res	res	res	F allmost fully erupted	E	C	F erupting	F	H	F erupted	C
	M369	res	res by I1	h	res	res	E	E	C	E	E	G	E	B
	M382	x	x	res by I2 and res by C	x	x	G erupted	F erupted	E	F erupting	F erupting	H	F erupted	D
	M403	res	res by I1	h	h	h	D	D	C	C	C	G	C	0
	M451	res	res by I1	h	res	res	E	D	C	D	D	F	D/E	B
	M453	x	res by I2 and res by I2		res	res	F erupted	E erupting	C	E	E	H	F erupted	C
	M454	x	x	h	res	res	F erupted	F erupted	E/D	F erupting	F erupting	H	F erupted	E / D
	M455	x	x	res	x	x	G /F	F erupted	D	F	F	G	F erupted	D
	M465	g (& res by I1)	f	e erupting?	g	f erupting	B	B	A	0	0	B/C	0	0
	M475 2nd	h	r	f	r	r	C	C	C	B	B	E	B	0
	M475A 1st	res	h	f erupting	h	g	C	C	C	A	A	C	0	0
	M506	x	x	x	x	x	H	H	H	H	H	H	H	H
	M507	x	res	h	res	res	F erupting	E	C	E	E	G	E	B
	M52	x	x	x	x	x	H	H	F	H	G	H	G	E
	M556	res	res by I1	g	res	h	C	C	C	B	A	E	B	0
	M60	res	h	h	h	res	E	E	C	E	E	G	E	B
	M635	x	res	res by I2	res	res	F erupting	E erupting	C	E	E	G	F erupted	C
	M636	x	x	res	res	x	H	H	D	F erupting	G erupting	H	G	E
	M674	res	res by I1	h	res	res	E	D	C	D	D	F erupting	D	0
	M744	res	h	h	res	res	E	E	C	E	E	G	E	B
	M781	res	res	F erupting	h	g	C	B-C	B	0	0	C	0	0
	M876	res	res by I1	h	res	res	E	E	C	E	E	G	E	C
	M911	res	h	h	res	res	E	E	C	E	E	F erupted	E / D	0
	M93	res	h	h	res	res	E	E	C	E	E advance P1	G	D	0/A
	M94	x	x	res	res	res	F erupted	E erupting	C	E	E	G	E	C
Gorilla gorilla														
	CAMII 351	h	h	f	h	h	C	C	C	C	C	E	A/B missing parts?	0
	M000	res	res	g	res	res	D	D	C	C	C	E erupting	B	0
	M109	x	x	x	x	x	H	H	E - F	mesial G, distal H	H	H	H	E
	M117	res	h	g	res	res	C	C	C	C	C	E	B	0
	M119 1st	x	res	res	res	res	E erupting	E	C	C	D	G	E	0
	M137	res	res	f	g	g	C	C	C	A	0	C	0	0
	M141	res	h	g	res	h	C	C	C	C	C	E	B	0
	M160	x	x	res by P1	res	res	E erupted	E erupted	C	E2	E1	G erupted	E	C
	M169	res	res	res	res	res	E	D	C	D	D	G erupted	E	A
	M177	x	x	x	x	x	H	H	H	H	H	H	H	H
	M180 1st	x	x	h but Res mesial side by I2	res	x	F	F	E	E erupting	F erupting	H	F erupted	D
	M22	x	x	res by P1	res	res	F	F	D	E	F erupting	H	F erupted	D
	M241	x	x	x	x	x	H	H	F erupting	G mesial root H distal root	H	H	H	F erupted
	M29	res	h	h	h	res	D	D	C	C	C	F erupting	C	0
	M319	res	h	h	h	h	D	D	C	C	C	F erupted	C	0
	M32	h	h	f	h	h	C	C	C	C	C	E	B	0
	M33	res	h	h	h	res	E	E	C	D	D	F erupted	D	A
	M333	res	h	g	res	res	C	C	C	C	C	E	C	0
	M34	res	h	g	h	h	E	E	C	D	D	F erupted	D	A
	M35	x	x	x	x	x	H	H	G	H	H	H	H	G erupting
	M387	x	x	res	x	x	G	F	E	E	F	H	F erupted	D
	M409	h	h	f	h	g	C	C	C	C	C	E	B	0
	M450	res	h	h	res	res	D	D	C	D	D	G	D	A
	M463	res	res	h	res	res	E	E	C	D	E	F erupted	E	0
	M471	res	h	g	res	res	C	C	C	C	C	E erupting	B	A
	M476	f erupted	e erupted	c	e erupting	e	B	B	A	0	0	A	0	0
	M487	res	h	h	res	res	C	C	C	C	C	F erupted	B	0
	M497	h	h	f erupted	h	h	C	C	C	C	C	E	A	0
	M532	res	res by I1	f erupted	h	h	C	C	C	C	C	E	B	0
	M625	res	res	h	h	res	E	E	C	D	D	F erupted	D	A
	M690 1st	h	h	f bifid	g mesial	g	C	C	C	C	B	D	A	0
	M756	g	f	d	f erupted	f erupting	C	C	A	0	0	C	0	0
	M887	f erupted	f erupted	d	f erupted	e erupting	C	C	A	0	0	C	0	0
	M98	res	h	h	res	res	E	D	C	D	D	F erupted	C/D	0
	M99	res	h	f	res	res	C	C	C	C	C	F erupting	B	0

Figure S1 Comparison of adult brain sizes with those published by Ashton & Spence [3] using a sample that included the one used here; females (f) and males (m) are shown separately. The species means given by [2] are indicated by stippled lines. The values for adult captive, i.e. provisioned, chimpanzees (>20 years) from Yerkes [4] are also shown. The numbers at the bottom indicate the sample sizes; for the present analyses specimens with M3 nearly in full functional occlusion were considered adult. The subsample used in the present study is evidently different, although statistical tests cannot be carried out because of lack of primary data in [2].

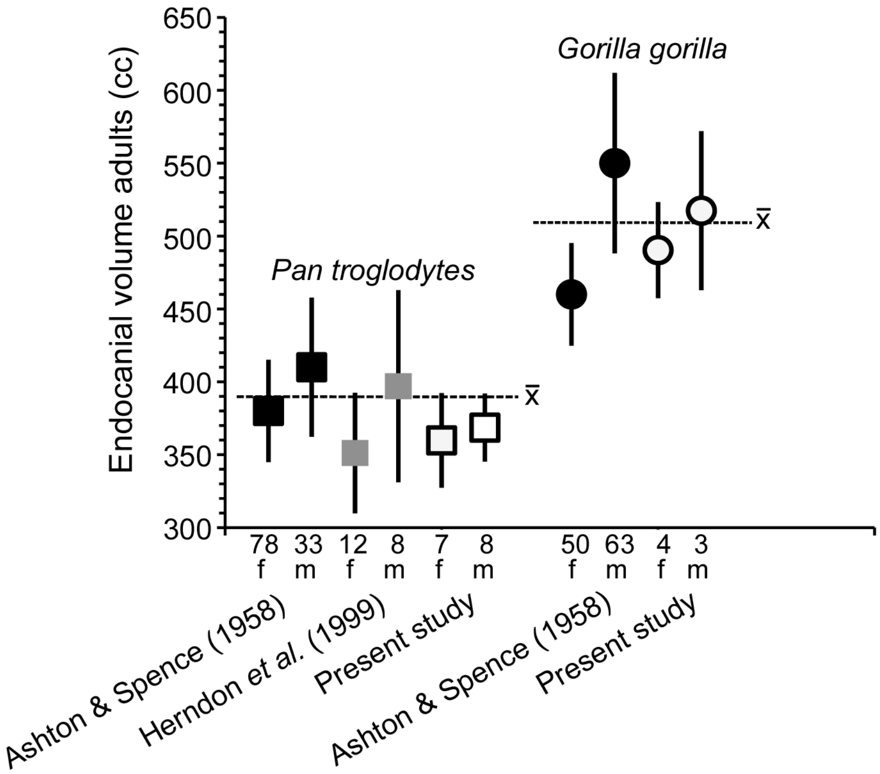

Table S3 Comparisons of actual data with those obtained through resampling with replacement (n = 1000). Results of Student´s *t*-tests are shown.

	Mean value Actual	Mean value Resampled	Std	Median	*t*-value	*p*
**Adult Endocranial volume ECV (cc)**						
$Gorilla_{female}$	490.37	490.54	14.74	488.76	0.0232	0.9815 *n.s.*
$Gorilla_{male}$	517.41	516.97	25.57	515.67	-0.0299	0.9761 *n.s.*
Pan_{female}	359.84	359.97	11.22	355.97	0.0309	0.9754 *n.s.*
Pan_{male}	368.57	368.59	7.36	365.81	0.0861	0.9978 *n.s.*
**Relative ECV (%)**						
Before M1 in functional occlusion						
Gorilla	87.76	87.72	1.88	87.83	-0.0446	0.9645 *n.s.*
Pan	sample size (n=2) too small to be meaningful					
After M1 in functional occlusion						
Gorilla	90.96	91.02	2.90	91.58	0.0716	0.9429 *n.s.*
Pan	96.40	96.49	2.58	96.25	0.1338	0.8936 *n.s.*

Table S4 Descriptive statistices for endocranial volumes by age group, sex and species.

			Age categories				
			Group 1	Group 2	Group 3	Group 4	Group 5
Gorilla gorilla							
	Females						
		Mean	414.76	442.98	456.19	476.75	531.24
		Std	26.99	4.23	68.07	22.66	
		N	3	3	4	3	1
	Males						
		Mean	443.70	471.36	492.91	489.15	517.41
		Std	67.38	40.81	48.36	34.09	
		N	8	3	7	2	1
Pan troglodytes							
	Females						
		Mean	321.52	334.55	315.13	358.41	363.42
		Std	37.03		32.60	39.67	0.82
		N	4	1	5	5	2
	Males						
		Mean	330.34	348.72	381.38	365.75	377.01
		Std	23.42		34.20	24.26	26.04
		N	4	1	10	6	2

Figure S2 Results of resampling procedure for $\delta^{15}N$ and $\delta^{13}C$ between infants (blue), juveniles (red) and adolescents/adults (green), for *Gorilla gorilla* and *Pan troglodytes* separately.

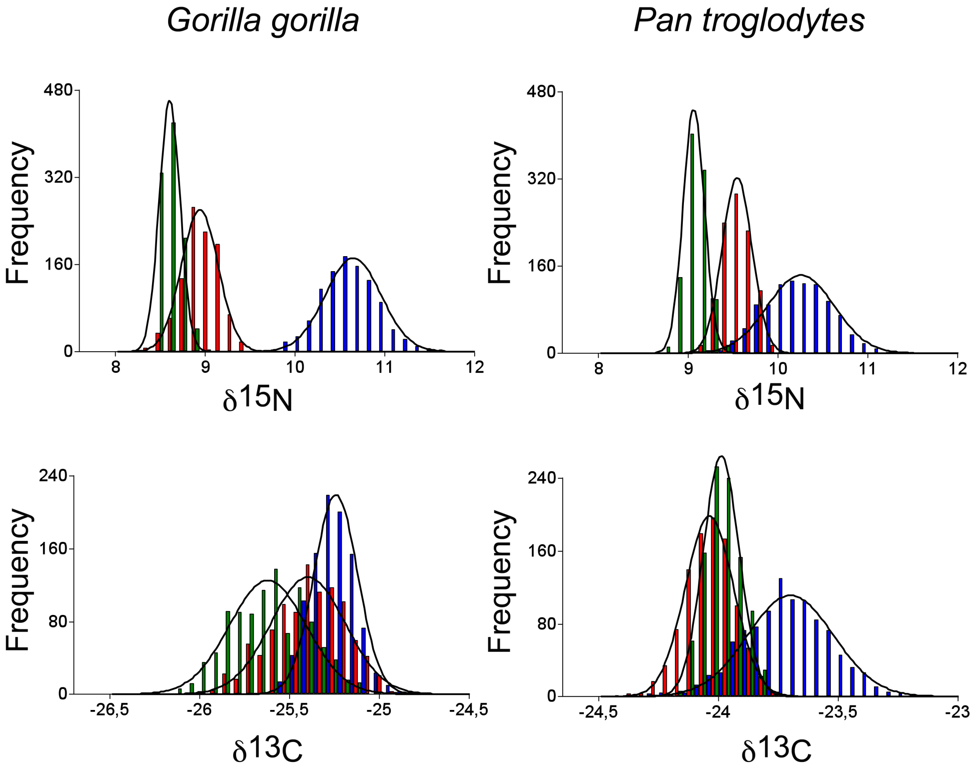

References

1. Wilson A, Taylor T, Ceruti M-C, Chavez JA, Reinhard J, Grimes V, Meier-Augenstein W, Cartmell L, Stern B, Richards MP, Worobey M, Barnes I, Gilbert MTP (2007) Stable isotope and DNA evidence for ritual sequences in Inca child sacrifice. Proc Natl Acad Sci USA 104 (42) 16456-16461.
2. Coquerelle M, Bayle P, Bookstein, FL, Braga J, Halazonetis DJ, Katina S, Weber GW (2010) The association between dental mineralization and mandibular form: a study combining additive conjoint measurements and geometric morphometrics. J Anthrop Sci 88: 129-150.
3. Ashton EH, Spence TF (1958) Age changes in the cranial capacity and foramen magnum of hominoids. Proc Zool Soc Lond 130: 169-181.
4. Herndon JG, Tigges J, Anderson DC, Klumpp SA, et al. (1999) Brain Weight Throughout the Life Span of the Chimpanzee. J Comp Neurol 409: 567-572.
